# Current Advances in Genetic Testing for Spinal Muscular Atrophy

**DOI:** 10.2174/0113892029273388231023072050

**Published:** 2023-12-20

**Authors:** Yulin Zhou, Yu Jiang

**Affiliations:** 1 United Diagnostic and Research Center for Clinical Genetics, Women and Children’s Hospital, School of Medicine & School of Public Health, Xiamen University, Xiamen, Fujian 361003, P.R. China;; 2 Biobank, Women and Children’s Hospital, School of Medicine, Xiamen University, Xiamen, Fujian 361003, P.R. China

**Keywords:** Spinal muscular atrophy, genetic testing, screening and diagnosis, MassArray^®^, digital PCR, next-generation sequencing, long-reads sequencing

## Abstract

Spinal muscular atrophy (SMA) is one of the most common genetic disorders worldwide, and genetic testing plays a key role in its diagnosis and prevention. The last decade has seen a continuous flow of new methods for SMA genetic testing that, along with traditional approaches, have affected clinical practice patterns to some degree. Targeting different application scenarios and selecting the appropriate technique for genetic testing have become priorities for optimizing the clinical pathway for SMA. In this review, we summarize the latest technological innovations in genetic testing for SMA, including MassArray^®^, digital PCR (dPCR), next-generation sequencing (NGS), and third-generation sequencing (TGS). Implementation recommendations for rationally choosing different technical strategies in the tertiary prevention of SMA are also explored.

## 1 INTRODUCTION

Spinal muscular atrophy (SMA) is one of the leading genetic causes of infant and childhood mortality worldwide [1]. Biallelic loss-of-function mutations in the survival of the motor neuron 1 gene (*SMN1*, OMIM #600354) contribute to the principal reason for SMA. The homozygous deletion of *SMN1* exon 7 accounts for about 94% of SMA patients, while compound heterozygous, where a deletion on one allele and one subtle mutation on the other one is responsible for the rest cases [2]. The survival of the motor neuron 2 gene (*SMN2*, OMIM #601627) plays a crucial role in disease phenotype modification, and the disease severity shows an inverse correlation with the copy number (CN) of *SMN2* [3, 4]. The two *SMN* genes exhibit a high degree of homology, with a mere variation of 5 base pairs between them [5].

In 2001, the European Molecular Genetics Quality Network (EMQN) established practice guidelines for conducting molecular analysis in patient diagnosis, carrier detection, and prenatal diagnosis of SMA. These guidelines utilize the molecular epidemiological characterization of the disease [6] and outline the principles of various techniques, including single-strand conformation polymorphisms (SSCP), restriction enzyme digestion, linkage analysis, and competitive amplification. Meanwhile, the EMQN emphasized that the detection performance of any method aimed at quantifying the *SMN* copy number should be thoroughly validated in the context of genetic testing applications for SMA. A decade later, the American College of Medical Genetics and Genomics (ACMG) proposed technical standards and guidelines for SMA genetic testing [7]. In this guideline, the advantages and disadvantages of four common genetic analysis techniques, restriction fragment length polymorphism (RFLP), multiplex ligation-dependent probe amplification (MLPA), quantitative PCR (qPCR), and Sanger sequencing, are discussed in different scenarios of clinical application (Table **1**). Among these methods, MLPA is universally regarded as the gold standard for diagnosis of SMA or carrier screening so far [8].

Molecular detection techniques for SMA have rapidly developed in the past decade. Methodological enhancements have been derived from the development of traditional instruments and novel approaches stemming from the utilization of emerging platforms. For example, high-resolution melting (HRM) analysis [9-12] and probe-based melting curve analysis [13] have been developed on a variety of fluorescence quantitative PCR instruments, whereas the CNVplex approach was developed on the platform of capillary-electrophore equipment [14]. Encouragingly, a series of novel methodologies, developed based on emerging platforms, such as DNA mass spectrometry [15, 16], digital PCR (dPCR) [17-29], Next-generation sequencing (NGS) [30-40], and long-read sequencing [41, 42], have been gradually incorporated into SMA genetic analysis and have been advantageous over traditional methods. Here, we aimed to review the underlying rationale and report the detection performance of these novel genetic testing applications, thereby offering valuable reference information for conducting genetic tests for SMA in clinical laboratories.

## 2 INNOVATIVE ANALYSIS PLATFORMS AND METHODS USED FOR SMA GENETIC TESTING

### 2.1 MALDI-TOF Mass Spectrometry

Matrix-assisted laser desorption ionization-time of flight mass spectrometry (MALDI-TOF MS) has been employed in the field of biochemical analysis for over two decades [43]. In recent years, a MALDI-TOF MS system named MassArray^®^ has provided sensitive and rapid detection of single-nucleotide polymorphisms (SNPs) and has gained popularity in pharmacogenetics [44], tumor profiling [45], liquid biopsy [46], hereditary genetics [47], and methylation analysis [48]. The principle of MassArray^®^ for genotyping assays is that the designed oligonucleotide primers would anneal directly to the target DNA in front of the SNP, followed by extension by one base in the presence of all four dideoxy nucleotides (ddNTPs). Subsequently, this base is identified by the time it takes for the extension primer mass to traverse the entire flight tube [43, 49]. In 2019, Lin *et al.* [15] reported the use of the MassArray^®^ technique for SMA genetic testing. In this study, primers for PCR and single-base extension were designed to target the c.840 and c.1155 loci of the *SMN* gene. After successfully validating one positive case in 167 previously genotyped dried blood spot (DBS) samples with known *SMN* CN, three neonates with SMA were identified among 29,364 Chinese newborns. As the first report of the MALDI-TOF MS-based technique in SMA genetic testing, these results demonstrate the feasibility of its use in SMA newborn screening. However, the inability to simultaneously analyze *SMN2* copy number and carrier status were major limitations of their work. Jin *et al.* [16] combined competitive PCR and MALDI-TOF MS (termed MS-CNV) for simultaneous quantification of the copy numbers of *SMN1* and *SMN2* using a 4-plex reaction system. To determine the *SMN* CN, a two-step normalization strategy was performed. Of the 141 cases analyzed, 24 patients, as well as 22 carriers, were successfully identified. Notably, the cut-off values for *SMN* CN determination were not presented in this paper; however, the authors claimed that ambiguous values of *SMN2* CN were observed in a small subset of patients for MS-CNV due to inaccuracies in peak signal intensity measurement. In addition to these two reports, MALDI-TOF MS has also been employed in the study of methylation modification of *SMN2* [48]. Overall, SMA genetic testing based on the MALDI-TOF MS platform has not been widely used until now, and the high price of the platform instrument and the complexity of the experimental design are considered the main factors hindering its clinical application [16].

### 2.2 Digital PCR

**Fig. (1):**
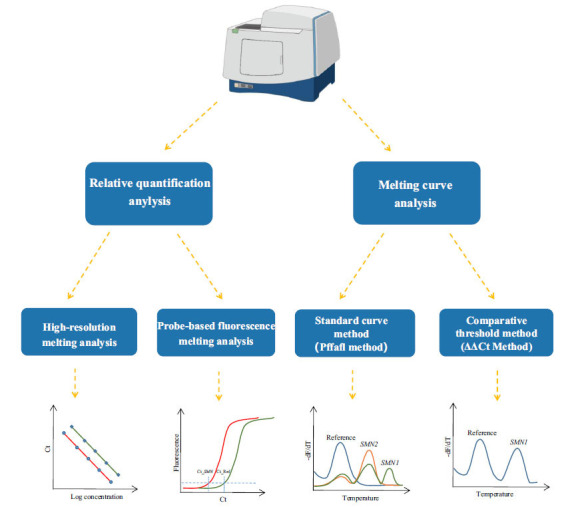
The analytical strategy used for *SMN* genes copy number detection on the qPCR platform. **Abbreviations:** qPCR, quantitative PCR; MLPA, Multiplex Ligation-dependent Probe Amplification; dPCR, digital PCR; NGS, Next-generation sequencing; HRM, High-resolution melting; CN, copy number; P/LP, pathogenic/likely pathogenic; Pos, positive result; Neg, negative result; SMA, spinal muscular atrophy, ECS, expanded carrier screening. Figdraw (www.figdraw.com) is used for figure plotting (Output ID: PPOAA533c6).

Owing to the continuous reduction in equipment costs and a wide range of application scenarios, real-time quantitative PCR (qPCR) instruments have become universally used in medical laboratories worldwide. Therefore, several SMA genetic testing methods have previously been developed based on qPCR platforms [9-13, 27, 50-57]. Collectively, the analytical strategy to detect *SMN* gene copy number using qPCR can be summarized as the following two main categories: relative quantification analysis based on ΔΔCT values and the normalized ratio of product peak area or peak height (Fig. **1**). Quantitative PCR-based approaches are more suitable as first-tier testing tools for patient diagnosis and newborn screening [9, 10, 27, 51] than carrier screening and *SMN2* copy number determination [54, 56]. The primary factor is the inherent challenge of precisely discerning various copy number combinations of *SMN* genes using non-absolute quantitative methods [12, 27, 51, 56].

In recent years, digital PCR (dPCR) instruments (also known as third-generation PCR) have been rapidly developed for molecular testing. Unlike qPCR, dPCR does not require a calibration curve to assign a numerical measure of copy number. DNA molecules are randomly distributed among tens of thousands of partitions in dPCR; therefore, copy number measurements are more precise and reliable than qPCR. Current digital PCR platforms are primarily based on microfluidic or microdroplet technologies (Table **2**).

Using a microdroplet-based rain dance droplet dPCR system, Zhong *et al.* [17] reported a pilot study using a single-tube 5-plex assay for SMA with FAM and VIC fluorophores with different fluorescence intensities to simultaneously measure the CN of *SMN1/2* exon7, and genotyping an SNP of *SMN1:*c.815A>G. As the first report on the application of the digital PCR method in SMA genetic analysis, the authors verified only one patient and four carriers and did not present cut-off values for *SMN* CN. Using a similar approach, Vidal-Folch *et al.* [21] developed a two-tube reaction digital PCR assay on a Bio-Rad QX200 Droplet Digital PCR system for the detection of *SMN1/2* copy numbers, as well as one SNP associated with the silent carrier. Using theoretical values as cut-offs for determining the *SMN* copy number and comparing the dPCR results of the validation set with the MLPA results, the authors obtained an accordance rate of 84.6% (14/17). In the verification sample set, digital PCR successfully identified 12 SMA-positive DNA samples and one SMA neonate from 1,530 DBS samples. In addition, the authors identified two individuals with potential silent carrier status among 125 subjects of Ashkenazi Jewish and other ancestries using a dPCR assay. Based on these results, the authors believed that they established a rapid and accurate approach to detect patients with SMA and increase the sensitivity of SMA carrier status. Park *et al.* [23] and Baker *et al.* [27] used the same platform for SMA genetic analysis using the accompanying commercial reagent for SMA genetic testing. The validation results of both studies were consistent with those of the reference method.

In 2015, Stabley *et al.* [18] employed a microfluidics-based array dPCR platform to detect CN in *SMN1/2* exon 7 and achieved a 100% concordance rate with the reference method for *SMN1* CN in 42 validation set samples. However, they found that the concordance decreased to 80% (12/15) for samples carrying more than two *SMN2* CNs in the validation set. Based on this observation, the authors suggested that dPCR could provide a more accurate measure of *SMN2* CN than qPCR. Subsequently, the authors established an assay to detect *SMN* gene conversion or partial deletion events by increasing the detection target [25]. With the improvement of the platform performance, Jiang *et al.* [22] developed a single-tube 4-plex reaction system to simultaneously detect *SMN1/2* exon 7 and intron 1 on the QuantStudio™ Absolute Q Digital PCR System. In contrast to the approach of Vidal-Folch *et al.* [21], Jiang *et al.* established a threshold based on the results obtained from ten control samples with known *SMN* copy numbers. A concordance rate of 93.3% (14/15) was achieved for the verification set. Another single-tube multiplex microdroplet dPCR assay for SMA genetic testing was conducted by Tan *et al.* [28] using the TD-2 Droplet Digital PCR System. The authors defined the extreme values obtained from 20 replicates of four samples with varying *SMN* copy numbers as cut-off values, resulting in an overall agreement rate of 95.9% (291/304). Meanwhile, the authors proposed that the droplet dPCR technique requires a smaller amount of DNA and yields more precise analysis outcomes than MLPA in the quantification of *SMN* copy numbers because 13 DBS samples could not be accurately detected by MLPA. Similarly, Wang *et al.* [26] reported a method for *SMN* copy number detection based on a chip-in-tube digital PCR platform by establishing a threshold range using extreme values from the validation cohort in 214 samples and obtained analytical results consistent with MLPA in 12 validation samples.

In addition to the aforementioned common samples, digital PCR has been preliminarily investigated for SMA genetic testing in non-traditional sample types. Recently, Gao *et al.* [29] employed a microdroplet-on-chip dPCR platform to develop a novel approach for detecting male SMA carriers using semen samples, including those from silent carriers. However, the authors also noted that relying solely on dPCR results cannot distinguish the “2+0” from the “1+0” genotype. To accurately differentiate between these genotypes, combining MLPA analysis of peripheral blood DNA with digital PCR analysis of sperm cells is recommended. This presents a significant constraint in this study. Additionally, semen is not commonly used as a sample source for large-scale SMA carrier screening. Thus, further evaluation of the clinical utility of this approach is required.

Over the past decade, there has been no shortage of reports detailing the implementation of digital PCR technology in the non-invasive prenatal diagnosis of monogenic genetic diseases [58-64]. In 2020, Wei *et al.* [24] developed a haplotype-free dPCR assay for the non-invasive prenatal diagnosis of SMA. Of the 92.6% (25/27) samples for which classifiable results were obtained, the *SMN1* copy numbers of fetal in maternal cell-free DNA were consistent with those obtained from amniotic fluid samples subjected to MLPA analysis. This report highlights the potential of dPCR for the non-invasive prenatal diagnosis of SMA; however, further studies with larger sample sizes are required before this strategy can be reliably applied in clinical settings.

### 2.3 Next-generation Sequencing

Over the past decade, next-generation sequencing (NGS) has been widely used to diagnose genetic diseases. Recently, NGS-based expanded carrier screening (ECS) has emerged as a potent tool for preventing birth defects caused by severe autosomal recessive (AR) and X-linked conditions [65]. However, the initial bioinformatics analytics model for NGS is often inadequate for analyzing CNVs in highly-homologous sequences, such as *SMN* genes, and additional complementary methods, such as qPCR, are necessary for targeted SMA testing [66]. In 2015, Larson *et al.* [30] utilized a Bayesian hierarchical model to evaluate the probability of an individual being an SMA carrier based solely on their *SMN1/2* reads obtained from NGS data. This study demonstrated the initiation of *SMN* copy number analysis utilizing NGS data (Table **3**). Using this model, the authors effectively identified four carriers out of 71 validation set samples. Subsequently, 16 individuals with a high probability of being carriers and 109 individuals with possible carrier status were identified from the publicly available NGS dataset of 2,501 samples. In 2021, Zhao *et al.* [37] utilized a modified Bayesian hierarchical model for the SMA carrier screening of 10,585 diverse couples in China, obtaining an overall carrier frequency of 1.4% for the Chinese population. Subsequently, by parallel comparison, the authors proposed that NGS is relatively more reliable for *SMN1* gene copy number detection because its repeatability is higher than that of qPCR and has the lowest retest rate among MLPA, qPCR, and NGS [38].

In 2017, Feng *et al.* [31] developed a bioinformatics approach termed paralogous gene copy-number analysis by ratio and sum (PGCNARS) to detect SMA carriers using *SMN1/2* reads at six loci of interest, achieving detection rates ranging from 90.3% to 95.0% in five ethnic groups. Based on Feng’s bioinformatics strategies, Shum *et al.* [40] integrated an NGS assay into an existing newborn screening program and established a laboratory workflow for adding SMA to newborn screening panels. After obtaining unanimous results from 12 positive and four negative samples, the aforementioned method was utilized to screen 2,552 DBS samples; however, no positive cases were identified. 

In 2020, Ceylan *et al.* [32] validated an *SMN* target sequencing assay in eight pre-characterized samples and demonstrated a 97.5% concordance rate with the expected results for 39 out of the 40 data points evaluated. Subsequently, they examined an independent cohort of 80 clinically well-characterized samples from the Turkish population to identify carriers and affected individuals with SMA and achieved a perfect correlation rate of 100%. Similarly, Huang *et al.* [39] employed targeted NGS of *SMN* genes to verify *SMN1* copy numbers in 75 carriers selected from a pre-characterized cohort of 5,200 individuals using qPCR as well as ascertaining the distribution of copy numbers for the *SMN2* gene among these carrier individuals simultaneously.

In 2020, Chen *et al.* [33] developed an NGS-based approach for distinguishing the copy number of *SMN1/2* genes by analyzing the read depth and eight discerning reference genome variations. This study demonstrated a concordance rate of 99.8% for *SMN1* CN and 99.7% for *SMN2* CN compared to orthogonal methods, with a sensitivity of 100% for patients and 97.8% for carriers. In the same year, Liu *et al.* [34] presented a workflow for analyzing *SMN* copy numbers using uniquely mapped reads from both *SMN* exon 7 and the control region. The authors established cut-off values based on perfect matching results obtained from a validation cohort (n=104) and subsequently identified eight patients and 60 carriers with different *SMN* CN in the verification set (n=3,734). Furthermore, the authors conducted a comparative analysis between the proposed method and those developed by Larson *et al.* [30] and Feng *et al.* [31], who demonstrated that the proposed method exhibited a superior detection rate and lower incidence of false positives than the aforementioned approaches.

Tan *et al.* [35] provided evidence supporting the diagnostic effectiveness of incorporating *SMN1* and *SMN2* analyses into a comprehensive multi-gene panel designed for neuromuscular disorders. The validation assay was conducted on a cohort of 68 individuals who had been previously diagnosed with SMA by qPCR. The concordance rates for *SMN1* and *SMN2* CN between NGS and qPCR were 100% and 93% (63/68) within the validation cohort, respectively. Furthermore, subsequent analyses revealed that all five inaccurate results originated from qPCR.

Similar to dPCR, NGS has also been explored for non-invasive prenatal diagnosis of SMA. Chen *et al.* [67] utilized targeted capture sequencing, focusing on 2,011 single nucleotide polymorphisms (SNPs) within a 3-megabase interval surrounding the *SMN1* gene, to conduct a pilot study on non-invasive prenatal diagnosis of SMA. This approach allowed the construction of haplotypes associated with pathogenic variants in the proband and employed a relative-haplotype-dosage (RHDO) analysis strategy. The authors successfully performed non-invasive prenatal diagnosis in five pregnant women with a family history of SMA, and their findings were consistent with those obtained through invasive prenatal diagnosis.

### 2.4 Third-generation Sequencing

Although dPCR and NGS-based methods have demonstrated clinical utility in SMA genetic analyses over the past few years, they still exhibit limitations in localizing small variations and detecting silent carriers. Recently, Li *et al.* [41] introduced a third-generation sequencing (TGS)-based approach called Comprehensive Analysis of SMA (CASMA), which employs long-range PCR and TGS. In this study, the sensitivity and specificity of CASMA in identifying “1+0” SMA carriers were 100% and 99.2%, respectively. CASMA is anticipated to enhance the detection rate of SMA carriers from 91% to 98% because of its ability to identify minor variants and silent carriers. However, the authors stated that the reliance of CASMA on haplotype analysis presents challenges in accurately determining copy number in instances where both *SMN1* and *SMN2* have the same haplotype and a read ratio of 1:1, albeit in rare occurrences. A comparable analytical approach was employed by Chen *et al.* [42]. In this study, a high concordance rate of 99.2% (118/119) for *SMN1* CN and a perfect concordance rate of 100% (116/116) for *SMN2* CN were achieved using TGS. Also, this study identified two *SMN1* haplotypes that demonstrated a robust association with the silent carrier phenotype in African populations.

## 3 RECOMMENDATION OF TECHNICAL STRATEGIES FOR SPINAL MUSCULAR ATROPHY GENETIC TESTING

**Fig. (2):**
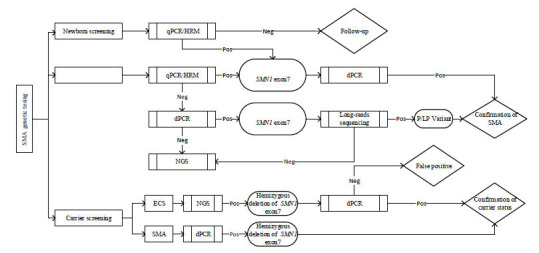
Recommendation of technical strategies for spinal muscular atrophy genetic testing. Recommended genetic testing strategies for SMA diagnosis, carrier screening, and newborn screening. **Abbreviations:** qPCR, quantitative PCR; MLPA, Multiplex Ligation-dependent Probe Amplification; dPCR, digital PCR; NGS, Next-generation sequencing; HRM, High-resolution melting; CN, copy number; P/LP, pathogenic/likely pathogenic; Pos, positive result; Neg, negative result; SMA, spinal muscular atrophy, ECS, expanded carrier screening.

Currently, the use of traditional methods, such as SSCP and RFLP, mentioned in the ACMG guidelines, has significantly declined in clinical practice. In addition to traditional methods based on qPCR or capillary electrophoresis, new SMA genetic detection approaches based on dPCR, NGS, and TGS have been developed and commercialized in recent years (Table **4**). However, there are significant differences in the cost of testing, turnaround time, types of variant detection, and testing performance between these methods. Given the plethora of platforms and approaches currently available for SMA genetic testing, selecting an appropriate test strategy for various application scenarios is crucial for reducing medical expenses, shortening turnaround times, and enhancing detection rates. Based on literature reports and our clinical experience, we herein recommend a laboratory test flow for SMA genetic testing in three distinct application scenarios (Fig. **2**).

The priority strategy for SMA newborn screening is to detect category 1 mutations (homozygous *SMN1* exon 7 deletion) using cost-effective, high-sensitivity, and high-throughput methods [7, 68]. Therefore, both qPCR platform-based probe-melting curve analysis [13] and the HRM technique [9-11] are suitable in this scenario. A positive result from newborn screening is recommended to be confirmed using a secondary method while simultaneously acquiring *SMN2* CN information [27, 69]. Currently, digital PCR, particularly single-tube multiplex reaction systems, is considered the most appropriate technique for the determination of *SMN2* CN [22, 28]. The recommendation of digital PCR over MLPA, which has traditionally been considered the gold standard for SMA diagnosis, is based on existing evidence suggesting the unsatisfactory accuracy of MLPA in detecting high *SMN2* copy numbers. In a retest of patients with SMA using the same MLPA assay for *SMN2* CN, Schorling *et al.* [70] found discordant results in nine of 20 (45%) cases. This issue was also observed in our practical experience.

A comparable strategy could be applied to genetically diagnose suspected SMA patients, where dPCR can verify positive findings from qPCR or HRM analysis and provide information on *SMN2* CN. If heterozygous deletion of *SMN1* is detected using dPCR in suspected SMA patients, long-read sequencing methods are recommended for detecting rare variants on the other allele [41, 42]. Otherwise, a differential diagnosis should be conducted using NGS [35].

Owing to the severity of the disease, professional organizations recommend periconceptional carrier screening for SMA in all couples, regardless of race or ethnicity [71]. For SMA carrier screening, both sensitivity and specificity are the key performance measures; therefore, methods with a high area under the curve (AUC) value from the receiver operating characteristic (ROC) curve should be considered first; second, cost, throughput, and turnaround time should be taken into account. To target a single disease for SMA, we recommend dPCR as the preferred method because of its high accuracy and low rate of false positives, whereas a positive result from NGS-based SMA carrier screening should be verified by a confirmatory test because of its relatively low sensitivity and higher false-positive rates compared to dPCR [37].

## CONCLUSION

In this review, we summarize the recent technological innovations in genetic testing for SMA. Despite the strengths of high throughput, few laboratories currently use the MALDI-TOF MS platform for the clinical application of SMA genetic testing because of the high cost of instrumentation and complexity of the experimental design. Digital PCR technology, owing to its inherent feature of absolute quantification, exhibits the highest resolution for determining *SMN* CN among currently available technologies and does not require a complex experimental design. Consequently, it is deemed suitable as a primary technique for SMA carrier screening as well as for validating and quantifying *SMN2* CN in patients with homozygous deletions. Currently, NGS technology is widely available and represents an optimal approach for implementing ECS, as well as a powerful tool for detecting minor variants and performing patient diagnoses of neuromuscular disorders, including SMA. However, given the inherent limitations of short read lengths, a complementary approach is necessary to confirm positive testing results. Compared to NGS, long-reads-based strategies have a natural advantage in the analysis of complex nucleic acid structures [72-74]. However, its high cost and relatively low throughput limit its application in large-scale population screening, making it more appropriate as a second or third-tier analysis for patients suspected of having SMA.

Although new technologies for the genetic testing of SMA are constantly emerging, it is crucial to emphasize the importance of performance validation procedures before these technologies can be implemented in clinical settings. It is worth noting that all the aforementioned novel approaches, akin to MLPA and qPCR, employ semi-quantitative methodologies to determine the copy numbers of *SMN* genes. Following the ACMG guidelines [7], it is imperative for laboratories conducting these tests to establish non-overlapping validated cut-off values, ensuring accurate and reliable differentiation of *SMN* copy numbers of 0, 1, 2, and 3. Meanwhile, the accuracy, precision, and confidence of *SMN* CN measurements around these established cut-off values should be known to the laboratory. However, most of the studies reviewed herein did not provide explicit methodologies for determining the cut-off values. We recommend that laboratories develop *SMN* CN quantification assays following the guidelines of the Clinical and Laboratory Standards Institute [75] to establish the cut-off value using receiver operating characteristic curve analysis. Performance validation, including accuracy, precision, and limit of detection metrics, is necessary before clinical application based on a defined cut-off value.

## Figures and Tables

**Table 1:** Comparison of traditional SMA genetic testing methods presented in ACMG standards and guidelines [7].

-	RFLP	MLPA	qPCR	Sanger Sequencing
Nature of Methodology	Qualitative Assay	Semi-quantitative Assay	Semi-quantitative Assay	Qualitative Assay
Patient Detection				
Category 1: [0+0]	++	+++	+++	+
Category 2: [0+1^d^]	-	+ ^a^	+ ^a^	++
Category 3: [1^d^ +1^d^]	-	-	-	+
Carrier Detection				
Category 1: [1+0]	-	+++	+++	-
Category 2: [2+0]	-	+ ^b^	+ ^b^	+ ^b^
Category 3: [1+1^d^]/[2+1^d^]	-	-	-	++
*SMN2* CN	-	++	+	-
Key quality control steps	Positive and negative controls should be included in each assay	1. ≥ Three reference samples should be included in each assay for data normalization; 2. ≥ One positive sample is recommended in each assay; 3. Requirement of high-quality DNA	1. Requirement appropriate internal reference gene; 2. Verification that the PCR amplification efficiency of the *SMN1* gene relative to the chosen internal reference gene is consistent between analyses	Verification that the intragenic mutation occurred in the *SMN1* gene and not the *SMN2* gene
Interpretation of Results				
Principle of result interpretation	Visual observation	Calculation of DQ after normalization of relative fluorescence intensity between the target gene and reference genes	Standard curve method, comparative threshold method, or melting curve analysis with visual observation	Visual observation
Cut-off values for *SMN* CN status determination	NA	Uniform cut-off values ^c^	Internal cut-off values obtained by validation experiments	NA

**Abbreviations:** ACMG, American College of Medical Genetics and Genomics; RFLP, Restriction fragment length polymorphism; qPCR, Quantitative PCR; MLPA: Multiplex Ligation-dependent Probe Amplification; CN: copy number; DQ: dosage quotient; NA: not applicable; *SMN1*: survival of motor neuron 1 gene; *SMN2*: survival of motor neuron 2 gene

^a^ Only testing *SMN1* copy number status; ^b^ Testing of two SNP loci, g.27134T>G and g.27706-27707delAT, which are considered associated with an increased risk of silent carriers; ^c^ See instruction protocol; ^d^ indicates minor variants.

The plus sign (+) denotes applicable, more plus signs indicate more appropriate, and the minus sign (-) denotes not applicable.

**Table 2:** Published literature on SMA genetic testing by digital PCR methods.

Study	Zhong *et al.*, 2011 [17]	Stabley *et al.*, 2015 [18]	Vidal-Folch *et al.*, 2018 [21]	Park *et al.*, 2020 [23]	Jiang *et al.*, 2020 [22]	Wang *et al.*, 2021 [26]	Stabley *et al.*, 2021 [25]	Baker *et al.*, 2022 [27]	Tan *et al.*, 2022 [28]	Gao *et al.*, 2023 [29]
Platform used (Type and number of partitions)	RainDance Droplet Digital PCR system (Microdroplet, 1,000,000)	QuantStudio™ 3D Digital PCR System (Microfluidics, 20,000)	Bio-Rad QX200 Droplet Digital PCR system (Microdroplet, 20,000)	Bio-Rad QX200 Droplet Digital PCR system (Microdroplet, 20,000)	QuantStudio™ Absolute Q Digital PCR System (Microfluidics, 20,000)	Clarity™ Digital PCR system (Chip-in-tube, 10,000)	QuantStudio™ 3D Digital PCR System (Microfluidics, 20,000)	Bio-Rad QX200 Droplet Digital PCR system (Microdroplet, 20,000)	TD-2 Droplet Digital PCR System (Microdroplet, 50,000)	BioDigital-QING dPCR™ system (Microdroplet-on-chip, 21,000)
Type of reaction system	Self-developed single-tube five-plex reaction system	Self-developed two-tube two-plex reaction system	Self-developed two-tube three-plex reaction system	Commercial two-tube two-plex reaction system	Self-developed single-tube four-plex reaction system	Commercial two-tube two-plex reaction system	Self-developed five-tube two-plex reaction system	Commercial two-tube two-plex reaction system	Commercial single-tube five-plex reaction system	Commercial single-tube three-plex reaction system
Fluorophores used and target sites	FAM: *SMN1/2* exon7 VIC: *SMN1*: c.815A FAM/VIC: *SMN1*: c.815A>G, *BCKDHA* (Ref)	FAM: *SMN1* exon7 or *SMN2* exon7 VIC: *RPPH1* (Ref)	FAM: *SMN1/2* exon7, *SMN1*: g.27134T HEX: *SMN1* exon7, *SMN1*: g.27134G, *RPP30* (Ref)	FAM: *SMN1* exon7 or *SMN2* exon7 HEX: *RPP30* (Ref)	FAM: *SMN1* exon7 VIC: *SMN2* exon7 TYE665: *SMN1/2* int1; TAMRA: *RPPH1* (Ref)	FAM: *SMN1* exon7 or *SMN2* exon7 HEX: *RPPH1* (Ref)	FAM: *SMN1* exon7 or *SMN1* exon8 or *SMN2* exon7 or *SMN2* exon8 or *SMN1/2* int1 VIC: *RPPH1* (Ref)	FAM: *SMN1* exon7 or *SMN2* exon7 HEX: *RPP30* (Ref)	FAM: *SMN1* exon7 ROX: *SMN1* exon8 VIC: *SMN2* exon7 CY5: *SMN2* exon8 CY5.5: *RPP30* (Ref)	FAM: *SMN1* exon7 HEX: *SMN2* exon7 ROX: *MRPS18C* (Ref)
Validation methods	NM	Quantitative capillary electrophoresis fragment analysis/qPCR/ sanger sequencing	MLPA	MLPA	QuantStudio™ 3D array dPCR assay	HRM/MLPA	MLPA	qPCR/ddPCR/sanger sequencing	MLPA	MLPA
Validation cohort and concordance rate	Patients: 4 Carriers: 1 Negative controls: 15 CR: 100%	Patients: 36 Carriers: 4 Negative controls: 2 CR: 100% for *SMN1* and 92.9% for *SMN2*	Patients: 2 Carriers: 5 Negative controls: 10 CR: 82.4% ^a^	NM	Patients:5 Carriers: 3 Negative controls: 2 CR:100%	Carriers:23 Negative controls:191 CR: 100%	Patients: 64 Unknown:30 Negative controls: 37 CR: NM	NM	NM	NM
Method for the cut-off values determination	NM	NM	Using the theoretical ranges	Embedded in the accompanying software	Using the mean ± 1.96SD ratio from the validation cohort	Using the extreme values from the validation cohort	NM	Embedded in the accompanying software	Using the extreme values from twenty replicates of four samples of different *SMN* gene copy numbers	NM
Study	Zhong *et al.*, 2011 [17]	Stabley *et al.*, 2015 [18]	Vidal-Folch *et al.*, 2018 [21]	Park *et al.*, 2020 [23]	Jiang *et al.*, 2020 [22]	Wang *et al.*, 2021 [26]	Stabley *et al.*, 2021 [25]	Baker *et al.*, 2022 [27]	Tan *et al.*, 2022 [28]	Gao *et al.*, 2023 [29]
Cut-off values	NM	NM	0 copy *SMN1/2* exon 7<0.5 0.5 ≤2 copy *SMN1/2* exon 7≤ 1.4 1.5 ≤2 copy *SMN1/2* exon 7≤ 2.4 2.5 ≤3 copy *SMN1/2* exon 7≤ 3.4 3.5 ≤4 copy *SMN1/2* exon 7≤ 4.4 4.5 ≤5 copy *SMN1/2* exon 7≤ 5.4	NM	NM	NM	NM	NM	0 copy *SMN1/2*≤ 0.03 0.41 ≤1 copy *SMN1/2*≤ 0.66 0.80 ≤2 copy *SMN1/2*≤ 1.20 1.32 ≤3 copy *SMN1/2*≤ 1.71 1.81 ≤4 copy *SMN1/2*≤ 2.21	NM
Verification cohort and analytical performance	NM	Patients: 63 Non-SMA controls: 40 CR: 100%	Patients: 12 Unknown: 1,530 DBS samples and 125 blood samples CR: 100% for patients; Detected potential silent carrier status in 2 individuals	Patients: 32 Carriers: 10 Negative controls: 158 CR: 100%	Patients:13 Negative controls: 2 CR: 93.3%	Carriers: 9 Negative controls: 3 CR: 100%	Patients: 12 CR: 100% for *SMN1* exon7/8, 91.7% for *SMN2* exon7, and 83.3% for *SMN2* exon8	Patients:6 CR: 100%	Patients:22 Carriers:72 Negative controls:223 CR: 95.9% ^b^	Carriers:17 Negative controls: 22 CR: 100% for controls and 88.2% for carriers

**Abbreviations:** DBS: Dried blood spots; Ref: Reference gene; HRM: High resolution melting; MLPA: Multiplex Ligation-dependent Probe Amplification; qPCR, Quantitative PCR; ddPCR, Droplet digital PCR; NM: Not mentioned; CR: Concordance rate; SD: standard deviation; int: Intron

^a^ The MLPA results of three samples were unable to be assigned a precise copy number due to their dosage quotient falling within a grey range according to the manufacturer’s instructions; ^b^ Thirteen DBS samples could not be accurately detected by MLPA due to low DNA concentration.

**Table 3:** Published literature on SMA genetic testing by NGS-based methods.

Study	Larson *et al.*, 2015 [30]	Feng *et al.*, 2017 [31]	Ceylan *et al.*, 2020 [32]	Chen *et al.*, 2020 [33]	Liu *et al.*, 2020 [34]	Tan *et al.*, 2020 [35]	Zhao *et al.*, 2021 [37]	Zhao *et al.*, 2022 [38]	Huang *et al.*, 2023 [39]	Shum *et al.*, 2023 [40]
Platform used	Illumina MiSeq	Illumina HiSeq 2500	Illumina NextSeq	Illumina HiSeq X/ HiSeq 2500/ NovaSeq 6000	Illumina HiSeq 2000/2500	NA	Illumina HiSeq 2000/4000	BIG MGISEQ-2000	Illumina NextSeq 500/550	Illumina NovaSeq 6000
Sequencing type (Sequencing depth)	Targeted sequencing (350×)	Targeted sequencing (300×)	Targeted sequencing (NM)	Whole genome sequencing (30x)	Targeted sequencing (220x)	Targeted sequencing (350x)	Targeted sequencing (>200x)	Targeted sequencing (NM)	Targeted sequencing (100×)	Targeted sequencing (300×)
Capture panel (Gene numbers)	Illumina TruSight Inherited Disease panel (552 genes)	Carrier screening panel (158 genes)	*SMN1* and *SMN2* genes	NA	Clinical exome panel (2,742 genes)	Neuromuscular disorders panel (122 genes)	Carrier screening panel (13 genes)	Carrier screening panel (237 genes)	*SMN1* and *SMN2* genes	Newborn screening panel (176 genes)
Variants types detection by NGS assay	*SMN1/2 CN*	*SMN1/2 CN,* *SMN1* g.27134T>G, *SMN* pathogenic variants	*SMN1/2* CN, *SMN1* g.27134T>G, g.27706-27707delAT	*SMN1/2* CN, *SMN2*Δ7–8, *SMN1* g.27134T>G	*SMN1* CN	*SMN1/2* CN *SMN* pathogenic variants	*SMN1* CN	*SMN1* CN	*SMN1/2* CN	Homozygous *SMN1*Δ7
Data analysis method	Bayesian hierarchical model	PGCNARS computational algorithm	Using normalized reads ratio value derived from GenePath Dx’s CODE-SEQ Dx(v1.02) analytics platform	Analyzing read depth and eight informative reference genome differences between *SMN1* and *SMN2*	Using a decision tree based on the thresholds for *SMN1* CN generated from the validation cohort	Comparing the depth of sequence coverage of *SMN1* and *SMN*2 with a calibrated set of baseline specimens	Bayesian hierarchical model	Bayesian hierarchical model	Normalizing read number and read number ratio of differences between *SMN1* exon7 and *SMN2* exon7	PGCNARS computational algorithm
Validation methods	qPCR/MLPA	qPCR/MLPA	MLPA	dPCR/MLPA	MLPA	qPCR/MLPA	qPCR	MLPA	qPCR	MLPA
Validation cohort and concordance rate	SMA Patients: 5 Non-SMA patient:6 Carriers: 4 unknown: 56 CR: 100%	Patients: 2 Carriers: 92 Negative controls: 6,648 CR: 100% for patients and 99.6% for carriers	Patients: 1 Carriers: 2 Negative controls:5 CR: 97.5% ^a^	Patients: 64 Carriers: 45 Negative controls:1,118 CR: 100% for patients, 97.8% for carriers	Patients: 17 Carriers: 69 Negative controls:18 CR: 100%	Patients: 68 CR: 100% for *SMN1* and 92.6% for *SMN2*	Patients: 1 Carriers: 72 Negative controls:2,182 CR: 100% for patients and carriers, 99.9% for controls	Patients: 16 Carriers: 133 Negative controls:329 CR: 100%	NM	Patients: 12 Negative controls: 4 CR: 100%
Verification cohort and analytic performance	Unknown:2,501 Detection of 16 high-probability carriers, 109 possible carriers, and 2,376 unlikely carriers	NM	Unknown:80 Detection of 18 patients and 21 carriers	Unknown:12,747 Detection of 251 carriers	Patients: 8 Carriers: 60 Negative controls:3,666 CR: 100% for patients, 90.9% for carriers	Unkown:5,304 Detection of 47 patients and 118 carriers	Unkown:20,968 Detection of 283 carriers and 85 possible carriers	NM	Carriers: 75 Negative controls:5,125 CR: 100% for carriers	Unkown:2,552 Specificity: 100%

**Abbreviations:** dPCR: Digital PCR; qPCR, Quantitative; MLPA: Multiplex Ligation-dependent Probe Amplification; CN: Copy number; PGCNARS: paralogous gene copy-number analysis by ratio and sum; CR: Concordance rate; NA: Not available; NM: Not mentioned.

^a^ 39 out of the 40 (97.5%) data points from 8 reference samples.

**Table 4:** Advantages and disadvantages of two mainstream and four novel techniques in genetic testing for SMA.

Approach	Advantages	Disadvantages
qPCR	1. High flux, low cost, easy to perform, and rapid 2. The popularity of qPCR instruments in medical laboratories is high	1. The accuracy for high *SMN2* CN quantification is low 2. For carrier screening, a calibration curve is required to rectify the difference in amplification efficiency between target and reference genes, which increases the testing cost
MLPA	1. The earliest commercialized kit for *SMN* CN quantification 2. Wide popularity, high acceptance	1. The capillary electrophoresis instrument is relatively expensive 2. Three reference samples are required in each assay for data normalization, which increases the testing cost 3. Sensitivity to contaminants
MALDI-TOF MS	1. High flux and low cost 2. Testing is rapid and cheap	1. The MALDI-TOF MS instrument is expensive 2. Complex experimental design and operating procedures 3. The accuracy for high *SMN2* CN quantification is low
dPCR	1. Results are robust and reliable 2. Testing cost is relatively low 3. Insensitivity to contaminants	Instrument accessibility and detection flux are lower than qPCR
NGS	1. High flux 2. Capable of simultaneous detection of *SMN* CN and minor variants 3. The testing cost is relatively low when SMA is screened as one of the target diseases in the ECS or NBS program	1. The NGS instrument is expensive 2. Complex experimental design and operating procedures 3. The accuracy for high *SMN2* CN quantification is low
TGS	1. Capable of simultaneous detection of *SMN* CN, minor variants, and silent carriers. 2. Highest detection rate	1. The TGS instrument is expensive 2. Complex experimental design and operating procedures 3. Highest testing cost

**Abbreviations:** qPCR, Quantitative PCR; MLPA: Multiplex Ligation-dependent Probe Amplification; MALDI-TOF MS: Matrix-assisted laser desorption ionization-time of flight mass spectrometry; dPCR: Digital PCR; NGS: Next-generation sequencing; TGS: Third-generation sequencing; SMA: Spinal muscular atrophy; *SMN2*: survival of motor neuron 2 gene; CN: Copy number; ECS: Expanded carrier screening; NBS: Newborn screening

## LIST OF ABBREVIATIONS

ACMG: American College of Medical Genetics and Genomics
AUC: Area Under Curve
CASMA: Comprehensive Analysis of SMA
CN: Copy Number
CNs: Copy Numbers
CR: Concordance Rate
DBS: Dried Blood Spot
ddNTP: Dideoxy Nucleotides
ddPCR: Droplet Digital PCR
dPCR: Digital PCR
DQ: Dosage Quotient
ECS: Expanded Carrier Screening
EMQN: European Molecular Genetics Quality Network
HRMA: High-resolution Melting Analysis
Int: Intron
MALDI-TOF MS: Matrix-assisted Laser Desorption Ionization-time of flight Mass Spectrometry
MLPA: Multiplex Ligation-dependent Probe Amplification
Neg: Negative Result
NGS: Next-generation Sequencing
P/LP: Pathogenic/likely Pathogenic
PGCNARS: Paralogous Gene Copy-number Analysis by Ratio and Sum
Pos: Positive Result
qPCR: Quantitative PCR
RFLP: Restriction Fragment Length Polymorphism
RHDO: Relative Haplotype Dosage
ROC: Receiver Operating Characteristic
SD: Standard Deviation
SMA: Spinal Muscular Atrophy
*SMN1*: Survival of Motor Neuron 1
*SMN2*: Survival of Motor Neuron 2
SNPs: Single-nucleotide Polymorphisms
SSCP: Single-strand Conformation Polymorphism
TGS: Third-generation Sequencing
